# Analysis of Dietary Habits and Nutritional Status of Children with Down Syndrome in the Context of Lipid and Oxidative Stress Parameters

**DOI:** 10.3390/nu14122390

**Published:** 2022-06-09

**Authors:** Edyta Wernio, Anna Kłosowska, Agnieszka Kuchta, Agnieszka Ćwiklińska, Kornelia Sałaga-Zaleska, Maciej Jankowski, Przemysław Kłosowski, Piotr Wiśniewski, Jolanta Wierzba, Sylwia Małgorzewicz

**Affiliations:** 1 Department of Clinical Nutrition, Medical University of Gdansk, 80-211 Gdansk, Poland; sylwia.malgorzewicz@gumed.edu.pl; 2 Department of Pediatrics, Hematology and Oncology, Medical University of Gdansk, 80-211 Gdansk, Poland; anna.klosowska@gumed.edu.pl; 3 Department of Clinical Chemistry, Medical University of Gdansk, 80-211 Gdansk, Poland; agnieszka.kuchta@gumed.edu.pl (A.K.); agnieszka.cwiklinska@gumed.edu.pl (A.Ć.); kornelia.salagazaleska@gumed.edu.pl (K.S.-Z.); maciej.jankowski@gumed.edu.pl (M.J.); 4 Department of Endocrinology and Internal Medicine, Medical University of Gdansk, 80-211 Gdansk, Poland; przemek.k@gumed.edu.pl (P.K.); piotr.wisniewski@gumed.edu.pl (P.W.); 5 Department of Internal and Pediatric Nursing, Institute of Nursing and Midwifery, Medical University of Gdansk, 80-211 Gdansk, Poland; jolanta.wierzba@gumed.edu.pl

**Keywords:** nutritional status, Down syndrome, obesity, eating habits

## Abstract

**Introduction**: The risk of obesity in children with Down syndrome is high. Undoubtedly, proper nutrition plays an important role in the prevention of excess body weight and is associated with a reduction of metabolic complications. The aim of the study was to assess the problem of disturbances in the nutritional status and eating habits of children with DS. **Methods**: A total of 39 patients were included in the study. The nutritional status was assessed by anthropometric tests and Dual X-ray Absorptiometry. Eating habits were assessed using the Child Eating Behavior Questionnaire and the Food Frequency Questionnaire. Blood samples were taken to determine the oxidative stress and lipid parameters. **Results:** Obesity was recognized in 15% of subjects and 23% were overweight. Children that were overweight were characterized by higher levels of triglycerides, atherogenic index of plasma, and apoA2 and apoE levels. Fat mass, fat mass/height^2^ index, and visceral fat mass correlated with thiobarbituric acid reactive substances and advanced oxidative protein product level. The analysis of the Child Eating Behavior Questionnaire showed that children struggling with being overweight were more interested in food compared to those with normal body weight. A positive correlation was identified between waist circumference and food interest categories. Insufficient consumption of dairy products, vegetables, whole grain products, as well as fruits, seeds, nuts, and fatty fish was noted. Patients were less likely to consume products that are a good source of mono- and polyunsaturated fatty acids. **Conclusions:** In children with Down syndrome and obesity, disturbances in lipid and oxidative stress parameters are observed. Abnormal eating habits in all children with Down syndrome regardless of their nutritional status were noted. Proper nutritional education, nutritional control, and management of metabolic problems are essential in this group of patients.

## 1. Introduction

Down syndrome (DS) is the most common chromosomal condition, affecting approximately one in every 1000 live births in Europe and one in every 792 live births in the USA [1]. The average life expectancy for people with DS has increased significantly, from 25 years in 1983 to 60 years in 2020 [2].

Recent studies also show that similarly to the general population, an increasingly common problem in DS subjects can be obesity and being overweight, even in the child population. According to O’Shea et al., 51.6% of boys and 40% of girls were overweight/obese [3]. Obesity can be associated with many health conditions. In youth with DS, the relationship between obesity with health disorders is difficult to establish because of many comorbid diseases attributed to DS. However, there are studies linking the weight status of youth with DS with adverse health outcomes such as dyslipidemia, hyperinsulinemia, obstructive sleep apnea, orthopedic and biomechanics complications, and impaired cardiorespiratory fitness.

The causes of abundant body mass in addition to genetic predisposition are also inappropriate lifestyle behaviors [4]. It was found that children with DS consume too much energy for their needs as well as lead a sedentary lifestyle [5]. It was also proven that educating parents of children with DS regarding nutrition can reduce the risk of obesity [6]. 

The presented preliminary study aimed to assess the nutritional status of children with DS and to determine their nutritional choices and behavior in the context of their body composition as well as lipid-related and oxidative stress markers.

## 2. Material and Methods

A total of 39 patients (15 boys, 24 girls) with Down syndrome were included in the study. All subjects were regular patients of the Pediatric Clinic at the University Clinical Center in Gdansk (Poland). The University Ethics Committee’s approval for the study was obtained (number NKBBN/105-136/2018., 12/04/2018).

The following inclusion criteria were set:

- Age ≥ 9 years and <18 years,
- Signed informed child–carer consent for participation in the study,
- No obesogenic drugs in the medical interview,
- Lack of severe concomitant diseases,
- A willingness to cooperate.

Exclusion criteria included:

- Mosaic Down syndrome,
- No consent for the study,
- Steroid treatment or other drugs affecting body weight,
- Severe associated diseases.

### 2.1. Anthropometry and Body Composition

The anthropometric measurements of each subject were performed in the fasting state during a follow-up visit after signing the consent for testing. Waist circumference was measured in the horizontal plane midway between the lowest rib and the iliac crest. Body mass (kg) was measured using a mechanical column scale (Seca 711). A telescopic stadiometer (Seca 220) was used to assess height (cm). BMI was calculated as body weight (kilograms) divided by the square of body height (meters). For the recognition of nutritional status, national centile charts (CC) for BMI were used. Overweight was defined as a BMI ≥85 and <95 CC, obesity ≥ 95 CC, and underweight <5 CC. 

The DXA (Dual X-ray Absorptiometry) method was used to assess body composition. The total body scanning time was 5 to 7 min and was performed by radiologic technologists who were trained for these examinations. The examination was carried out by standard procedure and data on fat mass (%, kg), visceral fat mass (g), lean body mass (%, kg), and bone mineral content (BMC) were obtained.

### 2.2. Assessment of Dietary Habits

Validated in the European population Child Eating Behaviour Questionnaire (CEBQ) includes a list of 35 items that characterize children’s eating styles. All responses were grouped into eight factors divided into two dimensions: food interest (FI) (food responsiveness (FR), emotional overeating (EOE), enjoyment of food (EF), and desire to drink (DD)) as well as food avoidance (FA) (satiety responsiveness (SR), slowness in eating (SE), emotional undereating (EU), and food fussiness (FF)). The questionnaire contains the following questions, for example: “My child loves to eat” (EF); “My child eats more when he is anxious” (EOE); “My child eats less when he/she is tired” (EUE); “When given new foods, my child initially refuses them” (FF); “Given the opportunity, my child would spend the day constantly drinking (soft drinks or sweetened juices)” (DD); “My child feels full before finishing a meal” (SR); My child eats more and more and slower during a meal” (SE). Parents rated their child’s eating behavior on a five-point Likert scale (never, rarely, sometimes, often, always; 1–5) [7]. The questionnaire was translated by a certified translator cooperating with the Medical University of Gdansk (Poland). 

A modified and validated country version of the Food Frequency Questionnaire (FFQ) was used for assessing the frequency of consumption in 62 product groups representing 8 main food groups last year (sweets and snacks, dairy products and eggs, grain products, fruits, vegetables and seeds, meat and fish, beverages). Respondents have a choice of 6 categories of food consumption frequency: (1) never or rarely, (2) once a month or less often, (3) several times a month, (4) several times a week, (5) daily, (6) several times a day [8]. 

### 2.3. Biochemistry

The blood sample was collected in a fasting state by qualified medical personnel. Total cholesterol (TC) and triglycerides (TG) levels in serum were measured by standard enzymatic colorimetric tests (Pointe Scientific, Poland). High-density lipoproteins (HDLs) were isolated by precipitation of apolipoprotein B (apo B)-containing lipoproteins with *heparin*-manganese reagent and HDL cholesterol (HDL-C) was determined enzymatically. LDL cholesterol (LDL-C) concentration was calculated using the Friedewald formula. The atherogenic index of plasma (AIP) was calculated as a logarithmic transformation of the TG to HDL-C ratio. Apolipoproteins: B (ApoB), A1 (ApoA1), A2 (ApoA2), and E (ApoE)concentrations were determined using the nephelometric method with antibodies obtained from Siemens Healthcare Diagnostics (Eschborn, Germany) with a Behring laser nephelometer. The level of thiobarbituric acid reactive substances (TBARS) was analyzed by fluorescence spectroscopy using a modified thiobarbituric acid-reactive substance method [9]. Advanced oxidative protein product (AOPP) determination was performed based on spectrophotometric detection [10]. 

### 2.4. Statistical Analysis

Statistica 13.3 for Windows was used for statistical analysis. The distribution of variables was assessed with the Shapiro–Wilk test. Data are presented as the mean ± standard deviation (SD) or median and interquartile range. Depending on the data distribution, a Pearson or Spearman test analysis of correlations was conducted. The t-test or U Mann–Whitney test was used in a comparative analysis of the two groups. The two-way ANOVA was used to examine the influence of gender on observed differences of continuous variables between the studied group. χ^2^ Pearson’s test was carried out to evaluate the association between categorized variables. The criterion for statistical significance was *p* < 0.05.

## 3. Results

### 3.1. Anthropometry and Body Composition

The characteristic of the study groups is presented in Table 1. Excess body weight was observed in 38% of children (obesity was recognized in 15% of subjects and 23% were overweight). Among 15 boys, 4 (27%) had abnormal body mass, in a group of 24 girls 11 (46%) had excess weight. The normal weight and overweight/obesity groups did not differ statistically in their gender, age, height, and comorbid diseases (Table 1).

The overweight/obese children had significantly higher fat mass and visceral fat mass (Table 1) and these differences were observed regardless of gender (*p* = 0.420; *p* = 0.179 for fat mass and visceral fat mass, respectively). There were no statistically significant differences in the content of fat-free mass as well as the bone mineral mass between the patients with normal weight and excess body mass (Table 1). 

In girls, a significantly higher level than in boys was observed for fat mass (16.8 (14.7;24.7) vs. 12.3 (8.8;15.8), *p* = 0.047), percent of body fat (38.9 ± 7.3% vs. 26.5 ± 6.2%, *p* < 0.001) and fat mass/height^2^ index (8.3 (7.1;12.2) vs. 4.9 (4.4;6.6), *p* < 0.001). In men, a higher fat-free mass (38.0 ± 9.0 vs. 28.0 ± 5.4, *p* = 0.002) and bone mineral mass (1509 ± 377 vs. 1238 ± 327, *p* = 0.033) were observed. Visceral fat content and waist circumference did not differ depending on gender. 

### 3.2. Biochemistry

The average values obtained for the whole study group for lipid profile parameters were below the cut-off values of 190 mg/dL for TC, 130 mg/dL for LDL-C, 145 mg/dL for non-HDL-C, and 150 mg/dL for TG, respectively (Table 2) [11]. The results above these values were obtained in six (15%), five (13%), five (13%), and three (8%) children, respectively. The mean HDL-C level for the study group was above the cut-off value of 40 mg/dL (Table 2). Four children (10%) had HDL-C below this value.

The children who were overweight were characterized by higher levels of TG, atherogenic index of plasma (AIP), and apoA2 and apoE levels (Table 2). 

The TG level and AIP correlated significantly with BMI, waist circumference, fat mass, fat mass/height^2^ index, and visceral fat mass (Table 3). ApoE level correlated with BMI, fat mass, and fat mass/height^2^ index. Moreover, fat mass, fat mass/height^2^ index, and visceral fat mass correlated with TBARS and/or AOPP levels (Table 3). For the other analyzed biochemical parameters, no statistically significant relationships were found.

### 3.3. Assessment of Dietary Habits

Taking into account the Child Eating Behavior Questionnaire, the analysis showed that children struggling with being overweight were more interested in food compared to those with normal body weight, especially in the male subgroup (Table 4). Children who were overweight/obese also received fewer points in categories associated with food avoidance. In the case of these subclasses, a higher difference between normal weight and obese children was observed for girls (Table 4).

A positive correlation was identified between waist circumference and food interest categories: enjoyment of food, food responsiveness, and desire to drink (Table 5). There were negative correlations between the results of food avoidance categories and BMI, waist circumference, fat mass (kg), fat mass/height^2^, and visceral fat mass (g) (Table 5).

### 3.4. The results of the Food Frequency Questionnaire

The dietary pattern of children with Down syndrome deviates significantly from the recommendations, especially in the area of consumption of dairy products, vegetables, whole grain products, which should be eaten several times a day, as well as fruits, seeds, nuts, which should be eaten every day, and fatty fish (at least one time in a week). Patients were less likely to consume products that are a good source of mono- and polyunsaturated fatty acids (Table 6).

Among the study group, 61% declared daily consumption of vegetables (at least a portion) and 51% indicated daily consumption of fruits. Only 20.5% of children consume at least one natural dairy product per day, and 28% determined that whole-grain products were eaten daily (for all they chose five points in FFQ). In total, 18% of them reached for sweetened milk products every day. 

A total of 7% of children added sugar to meals several times a day (6 points in FFQ) and 23% did this every day (5 points in FFQ). Additionally, 7% declared a daily consumption of sweets. A total of 10% of respondents consumed sweets and sugar once a month or less often. Salty snacks were eaten every day only by one child. In total, 59% of them declared consumption of these products once a month or less often. Additionally, sweetened beverages were less commonly consumed (several times a day by one child, several times a week by 8%, several times a month by 10%, the rest of them drank these beverages once a month or less often).

The dietary preferences of the study group included consumption of products rich in saturated fatty acids (Table 7). They consumed red meat more frequently than fish, but less frequently than white meat. In addition, they chose potatoes more often than starchy products. No differences were apparent in the consumption of vegetables compared to fruits. Fruits were consumed more often than sweets. Additionally, fruit and vegetable juices were more readily chosen than sweetened drinks.

## 4. Discussion

In the presented study, the nutritional status of children with Down syndrome and their dietary habits were determined, and attention was paid to their link with lipid and oxidative stress parameters. The psychological aspects related to food consumption were also assessed. 

In the study group, almost 40% of the patients had excess body mass. This result is consistent with general trends. In the United States, the prevalence of children with DS being overweight and obese is estimated at 23% and 20.6%, respectively [12]. 

Research evaluating the nutritional status of children with DS in Poland is scarce. Wrzochal et al. attempted to assess the problem of nutritional disorders in preschool children with DS. In a group of 61 subjects that were 3–6 years old, 24% had an excess body mass [13]. In conclusion, national observational studies should be conducted to assess the problem related to the inadequate nutritional status of children with DS and additionally to determine the age at which the risk of developing a nutritional disorder increases. 

As expected, overweight children also had higher visceral fat mass and waist circumference compared to children with normal weight. Interestingly, there were no differences in the fat-free mass between groups according to BMI. It is known that obese patients often have not only higher levels of body fat but also lean body mass compared to slimmer children [14,15]. 

In our study, there were no differences in lean body mass depending on the nutritional status. Similar to the population of healthy people, obese patients were expected to have a higher content of lean body mass than normal-weight children. The world of science draws attention to problems that may interfere with the proper quality/quantity of muscle mass in children with DS highlighting mainly a lack of physical activity [16,17]. Pitchford et al. showed that obesity in children with DS negatively correlated with their daily physical activity [18]. The muscle mass may also be affected by insulin resistance and inflammation associated with adiposity [19]. Abdominal obesity leads to increased circulating fatty acids, associated inflammation, and ectopic triglyceride storage (in muscle and organs) [17]. 

Considering gender, it was observed that girls had more body fat and lower levels of fat-free mass than boys, which is consistent with the results of other reports [14,15]. In conclusion, there is a common gender differentiation in body composition in the studied children with DS. The percentage of adipose tissue in both genders was higher than in the healthy children in the country. According to Golec et., in the population of Polish children ages 7–17 years old, the median percent of body fat was 22% and 14.5%, respectively, in girls and boys. More than 40% of males had excess body mass, as well as 34% of female subjects [20]. 

Various factors were mentioned among the causes that promote the development of obesity in DS. For example, low lean body mass, decreased basal metabolism rate, leptin resistance, abnormal TSH pattern, and probably hypothalamic dysfunction [21,22,23,24]. Moreover, inappropriate eating habits and emotional under-, or over-consumption should not be neglected [21].

In the present study, the CEBQ questionnaire was used for the first time to investigate the parental perception of eating behavior of children with DS from a psychological perspective, paying attention to the influence of emotions such as happiness, worry, anger, irritation, fatigue, nervousness and anxiety. Consumption patterns (speed of eating) and individual attention to food (waiting for a meal, asking for food) were also studied. By analyzing the results of the Child Eating Behavior Questionnaire (CEBQ), it was found that obese children were more interested in food and showed more enjoyment in eating than children of normal weight. Emotional avoidance of food was observed in children with normal weight. Food avoidance attitude was associated with lower BMI, waist circumference, and fat mass. A positive association was detected between waist circumference and subclasses of food avoidance attitude: enjoyment of food, reactivity to food, and desire to drink. 

The CEBQ questionnaire is used to evaluate parents’ perceptions of their children’s eating habits. Ek et al. demonstrated that parental pressure to eat was associated with food avoidance on the CEBQ in children and was mainly due to parental concern about child weight gain [25]. Interestingly, the main reason for children’s food avoidance was parents’ dietary restrictions rather than the feeding approach itself [25]. Thus, it can be assumed that caregivers played an important role in the maintenance of normal weight in individuals with DS. There is no doubt that the personality-motivational profile and the behavioral phenotype of individuals with DS should also be considered in the context of their dietary choices, e.g., disobedience may also influence nutritional status and excess body mass (choosing foods forbidden by parents) [26,27].

Analysis of food intake frequency revealed a significant proportion of saturated fatty acids in the diet of patients with DS (in all, regardless of body weight) and an insufficient intake of natural sources of mono- and polyunsaturated fatty acids. Many other dietary errors were detected in the study population, including insufficient frequency of intake of dairy products, vegetables, fruits, fatty fish, and whole-grain products, increased frequency of intake of foods with a high glycemic index (refined cereal products, potatoes, sweetened dairy drinks). No differences were observed in the frequency of food intake in any of the categories according to nutritional status. The FFQ dietary tool was designed to assess the frequency of food intake and did not pay attention to the portion size. However, it should be also emphasized that the FFQ results correlate with nutrient intake [28]. In the case of obese DS patients, hyperphagia may be a clinical manifestation and should be considered a potential factor for nutritional status disorders [22]. The avoidance of foods containing beneficial fatty acids such as nuts, seeds, as well as raw vegetables, fruits, and whole-grain products apart from inappropriate dietary habits may be a consequence of chewing problems and/or gastrointestinal symptoms [21,22,29]. Unquestionably, factors such as obesity and poor eating habits influence the occurrence of dyslipidemia and metabolic disorders. It is known that typical dyslipidemia in obese individuals is related to an increased TG level [30]. In the present study, overweight children were also characterized by an elevated TG level which correlated with BMI and body fat content parameters. We did not observe a significant decrease in HDL-C and the major HDL apolipoprotein: apoA1 levels, which often accompany high TG levels. However, there was a significant increase in apoA2 level, which is the second most abundant protein of human HDL. The physiological functions of apoA2 in lipoprotein metabolism and obesity development are not fully elucidated, but it was shown that apoA2 overexpression exhibits marked hypertriglyceridemia, insulin resistance, increased adiposity, and increased atherosclerosis [31]

In our study, we also observed an increased level of apoE level in obese and overweight children that correlated with BMI and fat mass parameters. ApoE is an exchangeable apolipoprotein found in TG-rich lipoproteins and HDL, which plays many important functions crucial for normal lipoprotein metabolism, but it was also found that its accumulation is related to hypertriglyceridemia [32]. It is expressed in adipose tissue cells during adipogenesis [33]. A recent study also shows that high levels of both apoE and apoA2 were strongly inversely associated with the ability of lipoprotein lipase to hydrolyze TG in plasma—the process which plays a key role in hypertriglyceridemia development [34]. In our study, we also observed higher AIP levels in obese/overweight children with DS that correlated with BMI and waist circumstances. AIP is a novel index composed of TG and HDL-C that is used to quantify blood lipid levels and also, as an indicator of dyslipidemia, cardiovascular diseases, and obesity [35]. Adipose tissue, through a complex interplay between adipokines and various biochemical mechanisms, has a significant impact on systemic oxidative stress development [36,37]. In the presented study, we used the concentration of TBARS as a marker of lipid peroxidation, and the AOPP level as a marker of protein peroxidation. Both parameters are well known and commonly used as indicators of redox imbalance, and recent research indicates a link between TBARS and traditional cardiovascular risk factors such as inflammation, diabetes, and hypertension [38,39]. Our results showed a positive correlation between AOPP and TBARS with adipose tissue mass and/or visceral fat mass. These data can confirm the role of adipose tissue in oxidative stress development, which is consistent with other studies [40]. However, in our study, we found no significant differences between normal and overweight DS patients, which is an intriguing result and requires further research on the factors influencing oxidative stress in this group of patients as well as investigating additional oxidative stress markers.

Our study has some limitations. The study sample was relatively small which was related to the number of patients that are treated in our center and the number of parents and children that consented to the participation in the tests and blood collection. However, it should be noted that it is a preliminary study. Moreover, a possibly more accurate method of assessing food consumption should be used, notwithstanding that the application of the FFQ allows for reference to the national recommendations. Nevertheless, the use of methods for the evaluation of body composition, metabolic parameters, as well as eating habits, deserve attention and constitutes an important value of the presented work.

To summarize, we found that the obesity rate among children with DS is notable and in DS children with obesity, the disturbances in lipid and oxidative stress parameters are observed that correlated with the changes in body composition. Even at a young age, the risk of metabolic complications related to obesity, oxidative stress, and an abnormal lipid profile increases and it is necessary to monitor and manage metabolic abnormalities to reduce the risk of their complications. The presented research results also emphasize the importance of diagnosis of all possible factors in the development and prevention of nutritional disturbances in children with DS. Attention should be paid to maintaining adequate physical activity, taking into account the role of parents in controlling and creating children’s eating habits, including emphasizing the importance of the qualitative and quantitative composition of the diet, highlighting the intake of fatty acids, and having a look at the glycaemic index of carbohydrates.

Early nutritional education of families, caregivers, and children with DS, as well as constant/regular dietary control should be part of the overall patient care.

## Figures and Tables

**Table 1:** Characteristics of study groups and comparative analysis of body composition and anthropometric measurements in DS children with normal and excess body mass.

Parameters	All *n* = 39	Normal Weight *n* = 24	Overweight/Obesity *n* = 15	*p*-Value
Girls/boys	24/15	13/11	11/4	0.317
Age (years)	14.3 ± 2.4	14.4 ± 2.3	14.1 ± 2.6	0.873
Cardiac operation in the first year of life (n, %)	19 (49%)	13 (54%)	6 (40%)	0.389
Atrial septal defect/*ventricular septal defect* (n, %)	8 (21%)	6 (25%)	2 (13%)	0.450
Atrioventricular canal defect (n, %)	14 (36%)	9 (38%)	5 (33%)	0.792
Thyroid hormone replacement therapy (n, %)	35 (90%)	22 (92%)	13 (87%)	0.631
Thyroid Stimulating Hormone (TSH) mU/L	2.5 (2.05;3.17)	2.47 (2;3.4)	2.6 (1.7;3.17)	0.312
Weight (kg)	48.9 ± 13.1	43.1 ± 10.6	57.8 ± 11.9	<0.001
Height (m)	1.46 ± 0.11	1.48 ± 0.12	1.45 ± 0.11	0.442
BMI (kg/m^2^)	23.2 ± 4.3	20.6 ± 3.0	27.1 ± 3.0	<0.001
Waist circumference (cm)	75.8 ± 10.9	69.8 ± 7.5	85.4 ± 8.3	<0.001
Fat mass (kg)	16.7 ± 7.2	12.7 ± 3.6	22.9 ± 7.1	<0.001
Fat mass percentile	73.0 (44.5;90.0)	56.5 (35.5;73)	91.5 (81.5;94.5)	0.001
Fat mass (%)	33.6 ± 9.2	29.3 ± 6.4	40.3 ± 9.1	<0.001
Fat mass/Height^2^ (kg/m^2^)	7.2 (4.8;10.6)	5.1 (4.6;7.3)	10.7 (10.3;12.7)	<0.001
Fat mass/Height^2^ percentile	53 (41.5;85.5)	45 (30.5;53.0)	87 (76.5;91.5)	<0.001
Visceral fat mass (g)	252 (201;332)	229 (172;272)	331 (257;491)	0.002
Fat-free mass (kg)	32.2 ± 8.7	31.2 ± 9.0	33.7 ± 8	0.452
Bone mineral mass (g)	1353 ± 370	1328 ± 411	1392 ± 307	0.629
Bone mineral mass (z-score)	−1.28 ± 1.55	−1.42 ± 1.82	−1.1 ± 1.02	0.530

Data presented as mean ± SD or median (lower, upper quartile) depending on the distribution. Statistical significance when *p* < 0.05.

**Table 2:** Analysis of differences in biochemical parameters between children with normal weight and excess body mass.

Biochemical Parameters	All *n* = 39	Normal Weight *n* = 24	Overweight/Obesity *n* = 15	*p*-Value
TC (mg/dL)	170 ± 32	163 ± 31	181 ± 31	0.088
LDL-C (mg/dL)	101 ± 29	97 ± 27	109 ± 31	0.204
HDL-C (mg/dL)	54 ± 12	53 ± 12	54 ± 12	0.964
Non-HDL-C (mg/dL)	117 ± 30	110 ± 27	128 ± 32	0.065
TG (mg/dL)	70 (52;94)	61 (49;76)	94 (64;123)	0.007
AIP	−0.25 (−0.35;−0.08)	−0.30 (−0.36;−0.23)	−0.19 (−0.28;0.08)	0.016
ApoB (mg/dL)	66 ± 11	64 ± 10	67 ± 12	0.130
ApoA1 (mg/dL)	141 ± 21	140 ± 21	143 ± 20	0.617
ApoA2 (mg/dL)	32 (29;36)	30 (28;34)	36 (31;37)	0.023
ApoE (mg/dL)	4.06 (3.55;4.72)	3.77 (3.29;4.12)	4.64 (4.05;5.10)	0.011
TBARS (µmol/L)	1.83 ± 0.53	1.83 ± 0.59	1.83 ± 0.43	0.986
AOPP (µmol/L)	142 ± 38	133 ± 34	156 ± 40	0.069

TC—total cholesterol, LDL-C—LDL cholesterol, HDL-C—HDL-C cholesterol, non-HDL-C—non-HDL cholesterol, TG—triglycerides, AIP—atherogenic index of plasma, Apo—apolipoprotein, TBARS—thiobarbituric acid reactive substances; AOPP—advanced oxidation protein products; Data presented as mean ± SD or median (lower, upper quartile) depending distribution. Statistical significance when *p* < 0.05.

**Table 3:** Relationship between anthropometric measurements, body composition, and biochemical parameters.

	BMI	Waist Circumference	Fat Mass (kg)	Fat Mass/Height^2^ (kg/m^2^)	Visceral Fat Mass (g)
TG	0.405 (*p* = 0.011)	0.310 (*p* = 0.054)	0.357 (*p* = 0.041)	0.370 (*p* = 0.036)	0.216 (*p* = 0.234)
ApoE	0.515 (*p* < 0.001)	0.246 (*p* = 0.131)	0.417 (*p* = 0.016)	0.438 (*p* = 0.012)	0.280 (*p* = 0.120)
AIP	0.377 (*p* = 0.019)	0.399 (*p* = 0.012)	0.331 (*p* = 0.060)	0.266 (*p* = 0.140)	0.227 (*p* = 0.211)
TBARS	0.091 (*p* = 0.588)	−0.071 (*p* = 0.667)	0.344 (*p* = 0.050)	0.374 (*p* = 0.035)	0.341 (*p* = 0.056)
AOPP	0.176 (*p* = 0.288)	0.240 (*p* = 0.146)	0.283 (*p* = 0.109)	0.396 (*p* = 0.025)	0.377 (*p* = 0.033)

TG—triglycerides, ApoE—apolipoprotein E, AIP—atherogenic index of plasma, TBARS—thiobarbituric acid reactive substances; AOPP—advanced oxidation protein products. Statistical significance when *p* < 0.05.

**Table 4:** The mean score of CEBQ depends on percentiles of body mass index.

**Whole Study Group**	**All** ***n* = 39**	**Normal Weight** ***n* = 24**	**Overweight/Obesity** ***n* = 15**	***p*-Value**
Food interest (FI)				
Emotional overeating	1.85 ± 0.79	1.82 ± 0.77	1.90 ± 0.85	0.816
Enjoyment of food	3.66 ± 0.73	3.49 ± 0.65	3.92 ± 0.79	0.041
Food responsivness	2.62 ± 0.85	2.45 ± 0.72	2.87 ± 0.98	0.169
Desire to drink	2.93 ± 1.10	2.80 ± 1.23	3.11 ± 0.87	0.307
FI all categories (mean)	2.76 ± 0.58	2.64 ± 0.48	2.95 ± 0.68	0.150
Food avoidance (FA)				
Emotional undereating	2.12 ± 0.71	2.30 ± 0.75	1.87 ± 0.60	0.134
Satiety responsivness	2.32 ± 0.66	2.45 ± 0.71	2.12 ± 0.56	0.104
Slowness in eating	2.74 ± 0.52	2.88 ± 0.54	2.55 ± 0.42	0.066
Food fussiness	2.72 ± 0.34	2.76 ± 0.32	2.67 ± 0.36	0.805
FA all categories (mean)	2.48 ± 0.32	2.60 ± 0.32	2.30 ± 0.23	0.004
**Girls**	**All** ***n* = 24**	**Normal Weight** ***n* = 13**	**Overweight/Obesity** ***n* = 11**	***p*-Value**
Food interest (FI)				
Emotional overeating	1.78 ± 0.65	2.02 ± 0.78	1.55 ± 0.40	0.158
Enjoyment of food	3.53 ± 0.72	3.34 ± 0.60	3.73 ± 0.80	0.149
Food responsivness	2.36 ± 0.76	2.15 ± 0.59	2.58 ± 0.87	0.264
Desire to drink	2.50 ± 0.81	2.09 ± 0.60	2.91 ± 0.82	0.017
FI all categories (mean)	2.55 ± 0.48	2.40 ± 0.47	2.69 ± 0.46	0.115
Food avoidance (FA)				
Emotional undereating	2.17 ± 0.81	2.61 ± 0.75	1.73 ± 0.61	0.014
Satiety responsivness	2.38 ± 0.55	2.48 ± 0.61	2.27 ± 0.49	0.450
Slowness in eating	2.72 ± 0.54	2.87 ± 0.59	2.56 ± 0.45	0.224
Food fussiness	2.68 ± 0.38	2.74 ± 0.39	2.62 ± 0.39	0.742
FA all categories (mean)	2.49 ± 0.34	2.68 ± 0.30	2.30 ± 0.26	0.011
**Boys**	**All** ***n* = 15**	**Normal Weight** ***n* = 11**	**Overweight/Obesity** ***n* = 4**	***p*-Value**
Food interest (FI)				
Emotional overeating	1.95 ± 0.98	1.61 ± 0.74	2.88 ± 1.05 *	0.058
Enjoyment of food	3.85 ± 0.72	3.64 ± 0.68	4.44 ± 0.52	0.078
Food responsivness	2.99 ± 0.86 *	2.75 ± 0.74	3.65 ± 0.91 *	0.050
Desire to drink	3.56 ± 1.18 *	3.52 ± 1.31 *	3.67 ± 0.86	0.999
FI all categories (mean)	3.09 ± 0.59 *	2.88 ± 0.38 *	3.66 ± 0.75 *	0.031
Food avoidance (FA)				
Emotional undereating	2.05 ± 0.57	1.98 ± 0.62	2.25 ± 0.41	0.296
Satiety responsivness	2.23 ± 0.82	2.43 ± 0.82	1.69 ± 0.55	0.117
Slowness in eating	2.79 ± 0.51	2.89 ± 0.52	2.50 ± 0.38	0.214
Food fussiness	2.78 ± 0.26	2.77 ± 0.26	2.79 ± 0.28	0.999
FA all categories (mean)	2.46 ± 0.30	2.52 ± 0.32	2.31 ± 0.15	0.117

* vs. girls in the corresponding group. Statistical significance when *p* < 0.05.

**Table 5:** Relationship between CEBQ results and parameters of nutritional status.

	BMI	Waist Circumference	Fat Mass (kg)	Fat Mass/Height^2^ (kg/m^2^)	Visceral Fat Mass (g)
Food interest (FI)					
Emotional overeating	0.230 (*p* = 0.2)	0.115 (*p* = 0.5)	0.058 (*p* = 0.7)	−0.091 (*p* = 0.6)	0.035 (*p* = 0.8)
Enjoyment of food	0.334 (*p* = 0.047)	0.394 (*p* = 0.016)	0.186 (*p* = 0.3)	0.118 (*p* = 0.5)	0.334 (*p* = 0.071)
Food responsivness	0.217 (*p* = 0.2)	0.380 (*p* = 0.020)	0.140 (*p* = 0.4)	0.031 (*p* = 0.9)	0.246 (*p* = 0.2)
Desire to drink	0.161 (*p* = 0.3)	0.354 (*p* = 0.032)	0.069 (*p* = 0.7)	−0.130 (*p* = 0.5)	−0.027 (*p* = 0.9)
Food interest in all categories (mean)	0.244 (*p* = 0.2)	0.387 (*p* = 0.018)	0.065 (*p* = 0.7)	−0.125 (*p* = 0.5)	0.105 (*p* = 0.6)
Food avoidance (FA)					
Emotional undereating	−0.013 (*p* = 0.9)	−0.115 (*p* = 0.5)	−0.161 (*p* = 0.4)	−0.181 (*p* = 0.3)	−0.092 (*p* = 0.6)
Satiety responsivness	−0.280 (*p* = 0.1)	−0.214 (*p* = 0.2)	−0.404 (*p* = 0.024)	−0.277 (*p* = 0.1)	−0.450 (*p* = 0.013)
Slowness in eating	−0.415 (*p* = 0.012)	−0.373 (*p* = 0.023)	−0.388 (*p* = 0.031)	−0.372 (*p* = 0.043)	−0.261 (*p* = 0.2)
Food fussiness	−0.130 (*p* = 0.5)	−0.026 (*p* = 0.9)	−0.105 (*p* = 0.6)	−0.099 (*p* = 0.6)	0.066 (*p* = 0.7)
Food avoidance in all categories (mean)	−0.383 (*p* = 0.021)	−0.414 (*p* = 0.011)	−0.510 (*p* = 0.003)	−0.447 (*p* = 0.013)	−0.436 (*p* = 0.016)

Statistical significance when *p* < 0.05.

**Table 6:** The food consumption by children with Down syndrome concerning the recommendations.

	All (*n* = 39)		
		% of Children Who Met the Requirements (Ate Products Several Times a Day)	% of Children Who Never, Rarely, Once a Month or Less Often Consumed Nourishing Products ****
Products that should be eaten several times a day *	Vegetables	7%	8%
	Whole grain products	2.5%	20%
	Natural dairy products	7%	27%
Products that should be eaten every day **	Vegetable oils and/or seeds nuts	20%	Oils 5% Nuts/seeds 61%
	Fruits	51%	10%
At least one a week ***	Fatty fish	43.6%	56.4%

In the Food Frequency Questionnaire * several times a day (6), ** daily (5), *** several times a month (3) and several times a week (4), **** never or rarely (1), once a month or less often (2).

**Table 7:** The comparison of the frequency of eating the main group of food products.

	All	*p*	Obesity/Overweight	*p*	Normal Body Mass	*p*
Fruits vs. sweets	4.1 ± 1.2 vs. 2.9 ± 0.7	<0.001	4.3 ± 1.5 vs. 2.8 ± 0.6	0.01	3.9 ± 1 vs. 3 ± 0.8	0.002
Fruits vs. vegetables	4.1 ± 1.2 vs. 4.4 ± 1.2	0.18	4.3 ± 1.5 vs. 4.5 ± 1	0.78	3.9 ± 1 vs. 4.3 ± 1.2	0.12
* PUFA, MUFA sources vs. ** SFA sources	2.3 ± 0.6 vs. 3.1 ± 0.7	<0.001	2.25 ± 0.6 vs. 3 ± 0.6	0.01	2.4 ± 0.6 vs. 3.2 ± 0.7	<0.001
Whole grain products vs. refined grain products	3.5 ± 1.5 vs. 3.9 ± 1.4	0.22	3.7 ± 1.4 vs. 4 ± 1.4	0.72	3.3 ± 1.6 vs. 3.9 ± 1.4	0.23
Red meat vs. white meat	3.1 ± 0.8 vs. 3.6 ± 0.7	0.01	3.3 ± 0.4 vs. 3.7 ± 0.9	0.06	3 ± 0.9 vs. 3.6 ± 0.4	0.08
Red meat vs. fatty fishes	3.1 ± 0.8 vs. 2.3 ± 0.9	<0.001	3.3 ± 0.4 vs. 2 ± 0.8	0.001	3 ± 0.9 vs. 2.4 ± 0.8	0.06
Red meat vs. lean fishes	3.1 ± 0.8 vs. 2.5 ± 0.8	<0.001	3.3 ± 0.4 vs. 2.5 ± 0.8	0.01	3 ± 0.9 vs. 2.6 ± 0.7	0.03
Potatoes vs. group of groats, pasta, rice	3.9 ± 0.9 vs. 2.8 ± 0.9	<0.001	3.9 ± 0.9 vs. 3 ± 0.8	0.03	3.8 ± 1 vs. 2.7 ± 0.9	0.001
Fruit juices, nectars vs. sweetened beverages	3.5 ± 1.2 vs. 1.9 ± 1.1	<0.001	3.4 ± 1.4 vs. 2.1 ± 0.1	0.007	3.5 ± 1.2 vs. 1.9 ± 1.7	<0.001
Vegetable juices vs. sweetened beverages	2.5 ± 1.1 vs. 1.9 ± 1.1	0.04	2.7 ± 1.6 vs. 2.1 ± 0.1	0.2	2.4 ± 1 vs. 1.9 ± 1.1	0.12

* PUFA (polyunsaturated fatty acid), MUFA (monounsaturated fatty acid)—main source: vegetable oils, nuts, seeds, avocado; ** SFA (saturated fatty acid)—main source: butter, cheese, sour cream, eggs. Statistical significance when *p* < 0.05.

## Data Availability

The study did not report any data.
